# METAMVGL: a multi-view graph-based metagenomic contig binning algorithm by integrating assembly and paired-end graphs

**DOI:** 10.1186/s12859-021-04284-4

**Published:** 2021-07-22

**Authors:** Zhenmiao Zhang, Lu Zhang

**Affiliations:** Department of Computer Science, Hong Kong Baptist University, Hong Kong, SAR, China

**Keywords:** Contig binning, Assembly graph, Paired-end graph, Dead end, Multi-view label propagation

## Abstract

**Background:**

Due to the complexity of microbial communities, de novo assembly on next generation sequencing data is commonly unable to produce complete microbial genomes. Metagenome assembly binning becomes an essential step that could group the fragmented contigs into clusters to represent microbial genomes based on contigs’ nucleotide compositions and read depths. These features work well on the long contigs, but are not stable for the short ones. Contigs can be linked by sequence overlap (assembly graph) or by the paired-end reads aligned to them (PE graph), where the linked contigs have high chance to be derived from the same clusters.

**Results:**

We developed METAMVGL, a multi-view graph-based metagenomic contig binning algorithm by integrating both assembly and PE graphs. It could strikingly rescue the short contigs and correct the binning errors from dead ends. METAMVGL learns the two graphs’ weights automatically and predicts the contig labels in a uniform multi-view label propagation framework. In experiments, we observed METAMVGL made use of significantly more high-confidence edges from the combined graph and linked dead ends to the main graph. It also outperformed many state-of-the-art contig binning algorithms, including MaxBin2, MetaBAT2, MyCC, CONCOCT, SolidBin and GraphBin on the metagenomic sequencing data from simulation, two mock communities and *Sharon* infant fecal samples.

**Conclusions:**

Our findings demonstrate METAMVGL outstandingly improves the short contig binning and outperforms the other existing contig binning tools on the metagenomic sequencing data from simulation, mock communities and infant fecal samples.

**Supplementary Information:**

The online version contains supplementary material available at 10.1186/s12859-021-04284-4.

## Background

During long-term genetic evolution, animals, including humans, have formed complex ecosystems of symbiotic relationships with diverse microbes. The gut microbiome is a community with the highest microbial density in the human body, including thousands of microbial species mixed in varying proportions and constituting a dynamic system. Most gut microbes are difficult to be isolated and cultured in vitro. Metagenomic sequencing is designed to directly sequence a mixture of microbes and explore microbial compositions and abundances by data post-processing.

**Fig. 1:**
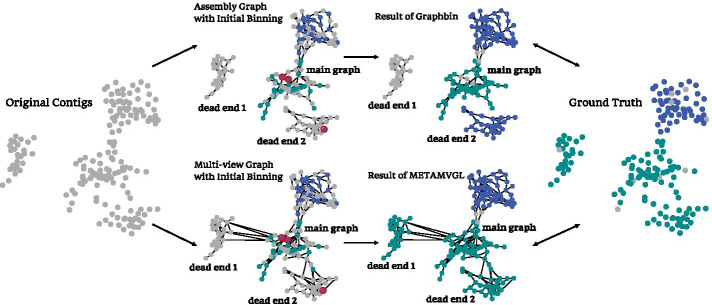
Visualization of the running process of METAMVGL compared with GraphBin in the simulated data. METAMVGL connected dead end 1 and 2 to the main graph by paired-end reads, also enhanced its connectivity. We observed (1) GraphBin failed to correct the two blue labels in the central of the graph, because it could not remove them before propagation due to lack of connectivity; (2) GraphBin mislabeled all the contigs in the dead end 2, caused by a small number of wrongly labeled contigs in the dead end; (3) METAMVGL labeled all the contigs in the dead end 1 but GraphBin did not

Due to the paucity of high-quality microbial reference genomes, current pipelines commonly target single genes or species using species-specific markers [1, 2]. But novel microbes would be lost by the alignment-based approaches. Metagenome assembly is a promising strategy to explore the novel species by concatenating the short-reads into contigs. But these contigs could be fragmented and can be only regarded as pieces of the target genomes. Contig binning algorithms provide a supplement to genome assembly that group the contigs into clusters to represent the complete microbial genomes. This strategy has been widely adopted to explore the novel microbes from the human gut metagenomic sequencing data [3–10].

Many state-of-the-art contig binning algorithms have been developed by considering contig nucleotide compositions (tetranucleotide frequencies (TNF), *k*-mer frequencies) and read depths. MaxBin2 [11] uses Expectation–Maximization algorithm to maximize the probability of a contig belonging to the local cluster centers using TNF and read depth. These two types of information are also used in MetaBAT2 [12] to calculate the contig similarities. MetaBAT2 constructs a graph using contigs as vertices and their similarities as the edges’ weights, which is further partitioned into subgraphs by applying a modified label propagation algorithm. CONCOCT [13] applies Gaussian mixture models for contig clustering based on *k*-mer frequencies and read depths across multiple samples. Besides considering TNF and read depths, MyCC [14] aggregates the contigs with complementary marker genes by affinity propagation; SolidBin [15] develops a spectral clustering algorithm using taxonomy alignments as must-links between contigs; BMC3C [16] applies codon usage in an ensemble clustering algorithm. All these methods are helpless in labeling short contigs (< 1 kb), because the limited number of nucleotides might lead to unstable TNF distributions and read depths. We observed a majority of the contigs (89.55%, Additional file 1: metaSPAdes assembly of *Sharon* dataset) in the assembly graph were shorter than 1 kb, which would be dropped by most of the existing binning algorithms.

**Fig. 2:**
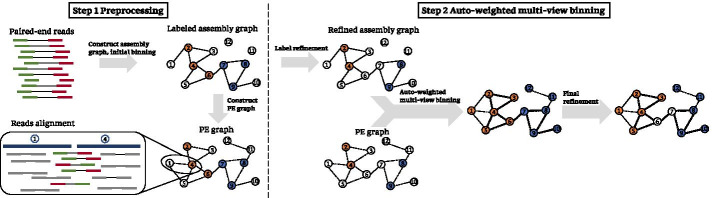
Workflow of METAMVGL. In step 1, METAMVGL constructs the assembly graph and PE graph by aligning paired-end reads to the contigs. The contigs are initially labeled by the existing binning tools (vertices in orange and blue). In step 2, the ambiguous labels are removed if their neighbors are labeled as belonging to the other binning groups in the assembly graph. METAMVGL applies the auto-weighted multi-view graph-based algorithm to optimize the weights of the two graphs and predict binning groups for the unlabeled contigs. Finally, it performs the second round ambiguous labels removal on the combined graph

To rescue those short contigs, Mallawaarachchi et al. developed GraphBin [17] to label the short contigs and correct the potential binning errors by employing label propagation on the assembly graph. In principle, the assembly graph should include *s* disconnected subgraphs, each representing one species. In practice, the subgraphs could be linked by the repeat sequences and some contigs are isolated from the main graph (the largest graph component) due to sequencing errors, imbalanced reads coverage, named dead ends. The performance of label propagation heavily relies on the number of edges and label density in the graph. The labels of short contigs would be significantly affected by the dead ends in two ways: (1) contigs are failed to be labeled if the dead end contains no label before propagation (Fig. 1 dead end 1); (2) erroneous labeling happens if only a small proportion of nodes are labeled in the dead end (Fig. 1 dead end 2).

**Fig. 3:**
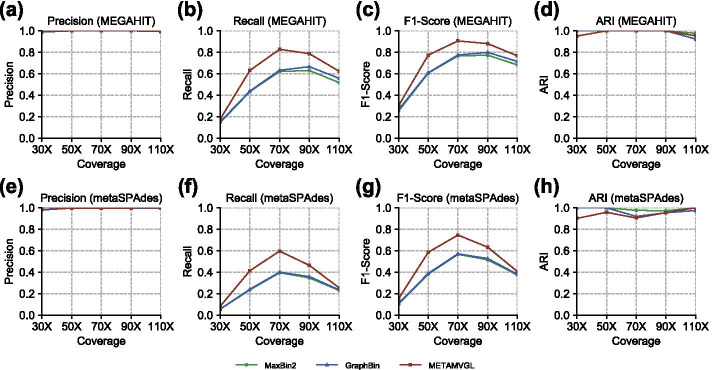
The performance of MaxBin2, GraphBin and METAMVGL on the simulated datasets. **a**–**d** Results based on the assembly by MEGAHIT, and **e**–**h** results based on the assembly by metaSPAdes. The initial binning tool is MaxBin2

**Fig. 4:**
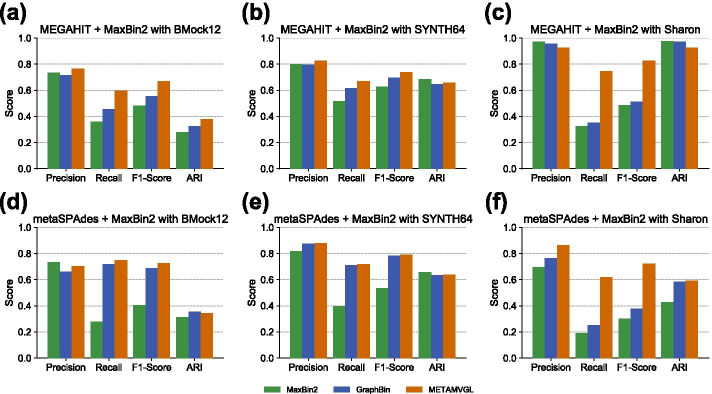
The performance of MaxBin2, GraphBin and METAMVGL on the *BMock12*, *SYNTH64* and *Sharon* datasets: **a**, **d** for *BMock12* dataset; **b**, **e** for *SYNTH64* dataset; **c**, **f** for *Sharon* dataset. MEGAHIT and metaSPAdes are used to generate the assembly graphs. The initial binning tool is MaxBin2

Here we present METAMVGL (Fig. 2), a multi-view graph-based metagenomic contig binning algorithm to address the above-mentioned issues. METAMVGL not only considers the contig sequence overlaps from the assembly graph but also involves the paired-end graph (PE graph), representing the shared paired-end reads between two contigs. The two graphs are integrated together by auto-weighting, where the weights together with the predicted contig labels are updated in a uniform framework [18] (*Methods*). Figure 1 gives a proof-of-concept example on the simulated data, where the paired-end reads connect the two dead ends (dead end 1 and 2) to the main graph. Our experiments indicate METAMVGL substantially improves the binning performance of the state-of-the-art algorithms, including MaxBin2, MetaBAT2, MyCC, CONCOCT, SolidBin and GraphBin in the simulated, mock and *Sharon* datasets (Figs. 3, 4, Additional files  3, 4, 5, 6: Figures). Comparing with assembly graph, the combined graph adds up to 8942.37% vertices and 15,114.06% edges to the main graph (Additional file 2: The assembly graph from MEGAHIT for* Sharon* dataset).

## Methods

Figure 2 demonstrates the workflow of METAMVGL, which consists of two steps. In step 1, METAMVGL constructs the assembly graph and PE graph with contig labels generated by the existing binning tools. In step 2, we remove the ambiguous labels of contigs if their neighbors are labeled as belonging to the other binning groups. The two graphs are integrated based on the automatic weights and the unlabeled contigs will be further predicted by label propagation on this combined graph. Finally, METAMVGL removes the ambiguous labels and generates the binning results.

### Step 1: Preprocessing

#### Construct assembly graph

We define the assembly graph as $\mathcal {G}_1(\mathcal {V}, \mathcal {E}_1)$, where the vertex $v_i \in \mathcal {V}$ represents the contig *i*, and an edge $e_{i, j} \in \mathcal {E}_1$ exists if $v_i$ and $v_j$ are connected in the assembly graph and with $k-1$ mer (continuous nucleotide of length $k-1$) overlap. In principle, the assembly graph should include *s* unconnected subgraphs, each representing one species and we can easily recognize contig binning groups. In practice, the subgraphs could be linked due to the inter-species repeat sequences and complicated by the sequencing errors and imbalanced genomic coverage. Commonly the assembly graph includes a main graph and several dead ends. Figure 2 illustrates an assembly graph with two dead ends (vertices 11 and 12). METAMVGL uses the assembly graph from metaSPAdes [19] or MEGAHIT [20]. The original assembly graph of metaSPAdes is a unitig-based graph, where each vertex represents a unitig. The contigs are sets of unitigs after resolving short repeats. Hence we convert the unitig-based graph to contig-based graph by adding the edge $e_{i,j}$, if at least two unitigs connect to each other and belong to $v_i$ and $v_j$, respectively. MEGAHIT would not provide the assembly graph directly, so METAMVGL uses contig2fastg module in megahit_toolkit to generate the graph in fastg format.

#### Construct PE graph

In order to deal with the dead ends in assembly graph, METAMVGL constructs the PE graph by aligning paired-end reads to the contigs by BWA-MEM[21]. For every two contigs $v_i, v_j$, we maintain a read name set $\mathcal {RS}_{i,j}$ to keep the names of read pairs, where the forward and reverse reads are aligned to the two contigs, respectively. The library insert size $\mathcal {IS}$ is calculated based on the uniquely aligned paired-end reads in the same contigs. To alleviate the influence of chimeric reads, METAMVGL links $v_i$ and $v_j$ if at least half of the reads in $\mathcal {RS}_{i,j}$ come from the two stretches with length $\mathcal {IS}$ in $v_i$ and $v_j$, respectively [22].

We denote the PE graph as $\mathcal {G}_2(\mathcal {V}, \mathcal {E}_2)$, where $\mathcal {V}$ represents contigs, and $\mathcal {E}_2$ the edges linked by the paired-end reads (PE links). According to our observation, PE graph is complementary to the assembly graph to some extent, because the edges in assembly graph ($\mathcal {E}_1$) only capture the overlaps between contigs, while the PE graph edges ($\mathcal {E}_2$) link the contigs with gaps. Figure 2 illustrates how dead ends of assembly graph can be linked to the main graph using PE links.

#### Initial binning

The contigs’ initial labels are generated by any existing binning tools. In experiments, we evaluated the performance of METAMVGL with the initial labels from MaxBin2, MetaBAT2, MyCC, CONCOCT and SolidBin in SolidBin-SFS mode. We used the default parameters for these algorithms except the MetaBAT2, where the minimum contig length was set to 1.5*kb* to label more contigs.

### Step 2: Auto-weighted multi-view binning

METAMVGL applies a multi-view label propagation algorithm [18] to learn the weights of assembly and PE graphs automatically and predict the unlabeled contigs in a uniformed framework. We remove the ambiguous labels for two times before and after label propagation.

#### Remove ambiguous labels

The initial contig labels could be incorrect especially for the ones from the inter-species repeat sequences and their influence would be amplified in label propagation. METAMVGL computes the distance between two vertices as the length of shortest path between them. Let $\mathcal {CLV}(v)$ be the set of labels from vertex *v*’s closest labeled neighbors in graph $\mathcal {G}$ and *v*’s label is ambiguous if $\mathcal {CLV}(v)$ contains a label that is different from *v* [17]. Let $\mathcal {VA}(\mathcal {G})$ denote the set including all the vertices with ambiguous labels in graph $\mathcal {G}$, and we remove the labels in $\mathcal {VA}(\mathcal {G})$. In Fig. 2, the closest labeled vertices of $v_6$ are $\{v_4, v_7\}$. Because $v_6$ and $v_7$ have different labels, the $v_6$’s label is marked ambiguous. Algorithm 1 shows the procedure to remove ambiguous labels. As shown in Fig. 2, we applied Algorithm 1 on the assembly and combined graphs before and after label propagation, respectively. We only use assembly graph to mark ambiguous labels in the preprocessing step for keeping more labels before propagation. 
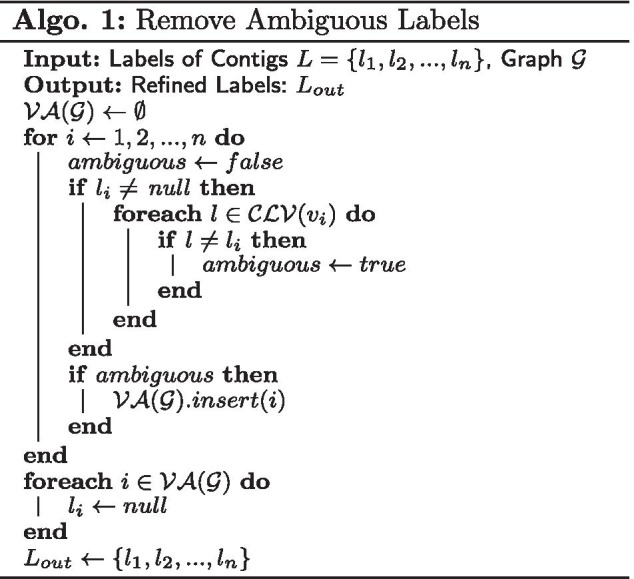


#### Auto-weighted multi-view binning algorithm

Assume *l* contigs are initially labeled with *s* groups, denoted as $Y_l=[y_1, y_2,\ldots , y_l]^T \in \mathbb {R}^{l \times s}$, where $y_{ij} \in \{0, 1\}$, and $y_{ij}=1$ indicates the contig $v_i$ is labeled from group *j*. We define a indicator matrix $F=[F_l;F_u] \in \mathbb {R}^{n \times s}$, where $F_l=Y_l$ and $F_u=[f_{l+1}, f_{l+2},\ldots , f_n]^T$ are labels to be inferred. Let $D_i, W_i \in \mathbb {R}^{n \times n}$ denote the degree and adjacent matrices of $\mathcal {G}_i$ ($i\in \{1,2\}$), respectively. The normalized Laplacian matrix of $\mathcal {G}_i$ has the formulation $\mathcal {L}_i = D_i^{-1/2}(D_i-W_i)D_i^{-1/2}$. According to [18], the inference of $F_u$ by label propagation can be modeled as the following optimization problem:

1 $$\begin{aligned} \underset{F_u}{\arg \mathrm {min}} \sum _{i=1}^{2} \sqrt{\mathcal {TR}(F^T\mathcal {L}_iF)},\ s.t.\ F_l = Y_l, \end{aligned}$$

where $\mathcal {TR}(\cdot )$ computes the trace of a matrix. The optimization problem is converted to

2 $$\begin{aligned} \underset{F_u}{\arg \mathrm {min}}\ \mathcal {TR}(F^T\mathcal {L}F),\ s.t.\ F_l = Y_l, \end{aligned}$$

where $\mathcal {L}=\sum _{i=1}^{2}\alpha _i\mathcal {L}_i$. $\alpha _i$ is the weight of $\mathcal {G}_i$, with initial values of 1/2. We partition $\mathcal {L}$ from $(l+1)$th row and column into four blocks as $[\mathcal {L}_{ll}, \mathcal {L}_{lu};\mathcal {L}_{ul},\mathcal {L}_{uu}]$. $F_u$ and $\alpha _i$ can be updated alternatively until convergence by the following equations [18]:

3 $$\begin{aligned} F_u&= \mathcal {L}_{uu}^{-1}\mathcal {L}_{ul}Y_l,\ F=[Y_l; F_u], \end{aligned}$$

4 $$\begin{aligned} \alpha _i&= \frac{1}{2\sqrt{\mathcal {TR}(F^T\mathcal {L}_iF)}},\ i\in \{1,2\}. \end{aligned}$$

Equation 3 can be considered as performing label propagation in the combined graph with iteratively updated weight $\alpha _i$, hence $\alpha _i$ implies the confidence of each graph. After obtaining $F_u$, we infer the labels of all the contigs by $l_i = \arg \mathrm {max}_{j}\ F_{ij},i\in \{1, 2, \ldots , n\}, j\in \{1,2,\ldots ,s\}$. Algorithm 2 shows the procedure of auto-weighted multi-view binning, and Fig. 2 is an illustration of this algorithm. 
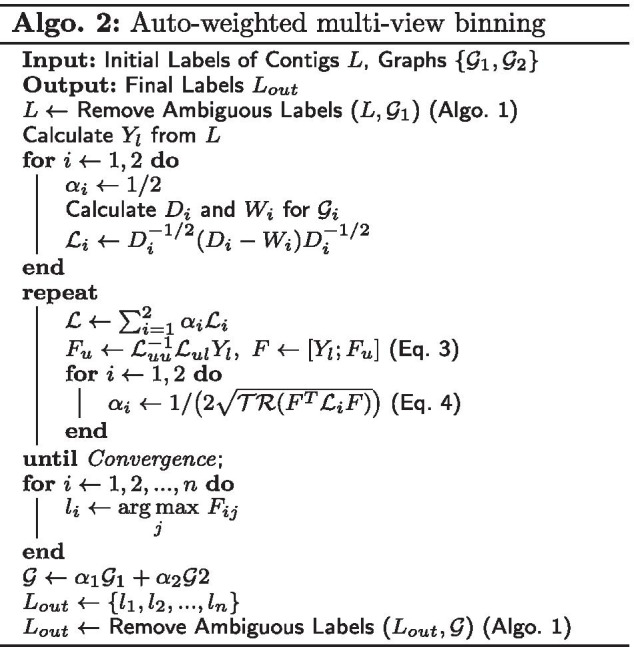


### Datasets

#### Simulated datasets

We simulated metagenomic sequencing data for a mixture of three strains with low, medium and high abundances. The components are:

- Acinetobacter baumannii: 0.90%,
- Streptococcus agalactiae: 9.01%,
- Streptococcus mutans: 90.09%.

We downloaded the complete reference genomes of the three strains from the NCBI Nucleotide Database (Taxonomy ID: 400667, 208435, 210007). CAMISIM [23] generated short-reads for the three strains with corresponding abundances. Five simulated datasets were generated with read depths as 30x (SIM_30x), 50x (SIM_50x), 70x (SIM_70x), 90x (SIM_90x) and 110x (SIM_110x).

#### Mock datasets

We evaluated the performance of METAMVGL on the metagenomic sequencing from two mock communities:

- *BMock12* refers to the metagenomic sequencing for a mock community with 12 bacterial strains sequenced by Illumina HiSeq 2500 [24] (NCBI acc. no. SRX4901583). It contains 426.8 million 150 bp short-reads with a total size of 64.4G bases.
- *SYNTH64* is a metagenomic sequencing dataset for a synthetic community with 64 diverse bacterial and archaea species [25] (NCBI acc. no. SRX200676), sequenced by Illumina HiSeq 2000 with read length 101*bp* and total size 11.1G bases.

#### Real dataset

*Sharon* dataset [26] (NCBI acc. no. SRX144807) contains the metagenomic sequencing data of infant fecal samples from 18 time points, sequenced by Illumina HiSeq 2000 with a total of 274.4 million 100 bp short-reads. We combined all the 18 datasets for co-assembly and referred them as *Sharon*.

### Evaluation criteria

We annotated the potential species the contigs came from as ground truth to compare METAMVGL with the other tools. For the simulated and mock datasets, we aligned the contigs to the available reference genomes and selected the ones with unique alignments. For the *Sharon* dataset, we used Kraken2 [27] to annotate the contigs according to *k*-mer similarities with the species from the build-in database.

Assume there are *s* ground truth species, and the binning result have *k* groups. To evaluate the binning result, we define the assessment matrix $[n_{i,j}]^{(k+1) \times (s+1)}$, where $n_{i,j}$ represents the number of contigs in *i*th binning group that are annotated *j*th ground truth species. The $(k+1)$th row denotes unbinned contigs. The $(s+1)$th column indicates contigs without ground truth annotations. We applied (1) Precision, (2) Recall, (3) F1-Score and (4) Adjusted Rand Index (ARI) to evaluate the performance of binning algorithms. Let $N = \sum \nolimits _{i=1}^{k} \sum \nolimits _{j=1}^{s} n_{i,j}$ be the number of contigs; the four metrics are calculated as follows:

1. Precision $$=\frac{1}{N}\sum \limits _{i=1}^{k} \underset{j \le s}{max}(n_{i,j})$$,
2. Recall $$=\frac{1}{N + \sum \limits _{j=1}^{s} n_{k+1,j}}\sum \limits _{j=1}^{s} \underset{i \le k}{max}(n_{i,j})$$,
3. F1-Score $$=\frac{2\times Precision \times Recall}{Precision + Recall}$$,
4. ARI $$=\frac{\sum \nolimits _{i=1}^{k} \sum \nolimits _{j=1}^{s} \left( {\begin{array}{c}n_{i,j}\\ 2\end{array}}\right) - t}{\frac{1}{2}\bigg (\sum \nolimits _{i=1}^{k} \left( {\begin{array}{c}\sum \nolimits _{j=1}^{s} n_{i,j}\\ 2\end{array}}\right) + \sum \nolimits _{j=1}^{s} \left( {\begin{array}{c}\sum \nolimits _{i=1}^{k} n_{i,j}\\ 2\end{array}}\right) \bigg ) - t}$$,

where $$t = \frac{1}{\left( {\begin{array}{c}N\\ 2\end{array}}\right) }\sum \nolimits _{i=1}^{k} \left( {\begin{array}{c}\sum \nolimits _{j=1}^{s} n_{i,j}\\ 2\end{array}}\right) \sum \nolimits _{j=1}^{s} \left( {\begin{array}{c}\sum \nolimits _{i=1}^{k} n_{i,j}\\ 2\end{array}}\right)$$.

## Results

METAMVGL was compared to six binning tools, MaxBin2, MetaBAT2, MyCC, CONCOCT, SolidBin in SolidBin-SFS mode and GraphBin. We analyzed their binning results on the five simulated datasets with various read depths, two mock communities (*BMock12* and *SYNTH64*) and a real metagenomic sequencing dataset (*Sharon* dataset).

### Evaluation on the simulated datasets

Figure 3 shows the binning results of the simulated datasets. The contigs and assembly graph were generated by MEGAHIT (Fig. 3a–d) and metaSPAdes (Fig. 3e–h). MaxBin2 was applied as the initial binning tool for GraphBin and METAMVGL.

All the three binning algorithms (MaxBin2, GraphBin and METAMVGL) yielded extremely high precision and ARI (Fig. 3a, d, e, h), due to the low complexity of the simulated datasets. Because of considering assembly and PE graphs jointly, METAMVGL labeled more contigs than GraphBin and MaxBin2 across various sequence depths, as shown in Fig. 3b, f. We also found both Recall and F1-Score were improved as read depth became higher until SIM_70x (Fig. 3b, c, f, g). This observation was analog to the results from CAMISIM [23], suggesting too high read depth would introduce assembly noise even it might help in detecting the low-abundance microbes.

### Evaluation on the mock communities

We illustrate the binning results for two mock communities with initial binning tool of MaxBin2 in Fig. 4a, b, d, e. In general, the graph-based methods (METAMVGL and GraphBin) were better than MaxBin2, but their performance would be influenced by the assembly graph. We observed the recalls could be significantly improved using the assembly graph from metaSPAdes (Fig. 4d, e), but the elevation became unobvious by the one from MEGAHIT (Fig. 4a, b). This was probably because metaSPAdes could generate more accurate and complete assembly graph than MEGAHIT. In the mock communities, METAMVGL was just slightly better than GraphBin, suggesting the PE graph was largely overlapped with the assembly graph (Additional file 2: *BMock12* and *SYNTH64* datasets). This observation only occurred if a perfect assembly graph was generated due to a low microbial complexity in the community. The results for the other initial binning tools (MetaBAT2, MyCC, CONCOCT and SolidBin) could be found in Additional files 3, 4, 5, 6: Figures, which were akin to the observations from MaxBin2.

### Evaluation on *Sharon* datasets

Figure 4c, f describe the binning results of MaxBin2, GraphBin and METAMVGL in *Sharon* dataset. METAMVGL substantially improved the recalls comparing with GraphBin (2.12 and 2.46 times on the assembly graph from MEGAHIT and metaSPAdes, respectively) and MaxBin2 (2.29 and 3.24 times on the assembly graph from MEGAHIT and metaSPAdes, respectively). METAMVGL showed the highest precision on the assembly by metaSPAdes (Fig. 4f). All the three binning algorithms were comparable in ARI.

The outstanding recalls from METAMVGL validate the capability of PE graph to connect the dead ends to the main graph when the assembly graph is incomplete in the complex microbial community. We observed that MEGAHIT produced very fragmented assembly graph in the *Sharon* dataset, in which the main graph only had 59 vertices with 64 edges, while a total of 15,660 vertices existed in the whole graph (Additional file 2: MEGAHIT assembly of *Sharon* dataset). The fragmented assembly graph was also mentioned as a limitation of GraphBin [17]. With PE links, METAMVGL yielded 5335 vertices and 9737 edges in the main graph (Additional file 2: MEGAHIT assembly of *Sharon* dataset), rescuing a large number of unlabeled contigs from the dead ends (Fig. 4c). Although the assembly graph was more complete (23.69% vertices in the main graph) from metaSPAdes, the PE graph still added 28.97% edges to the main graph (Additional file 2: metaSPAdes assembly of *Sharon* dataset) and improved the recall substantially.

## Discussion

De novo assembly together with contig binning offer a practical way to explore the novel microbes from metagenomic sequencing. But the current binning algorithms work stably merely on long contigs; the shorter ones are commonly neglected in the subsequent analysis. We observed a large proportion of contigs were shorter than 1 kb, which resulted in low completeness of the binning groups. A recent study [17] suggests the short contigs could be rescued from the assembly graph by considering their connections with the labeled ones. Assembly graph is accurate, but its connectivity relies heavily on the complexity of microbial community. Extremely low or high read depth, sequencing errors and imbalanced coverage could generate considerable dead ends, which would introduce both missing labels and labeling errors (Fig. 1).

In experiments, we observed a slightly lower ARI of METAMVGL comparing with MaxBin2 and GraphBin when read depth was low (Fig. 3d, h). It might because METAMVGL could only retrieve very few and low-confidence PE links. First, the label propagation would perform poorly on the contigs with low read depth. Comparing with GraphBin, METAMVGL included more edges in the graph, but these edges were sparse and cannot guarantee the good performance of label propagation, e.g. the unlabeled contigs might only have one neighbor. Second, the label refinement after label propagation could remove a majority of erroneous labels generated by METAMVGL based on our experience. Due to the paucity of edges, this step also performed inefficiently. Third, the quality of the contigs assembled from the sequencing data with low read depth was poor, making difficulties in aligning paired-end reads correctly.

In this paper, we developed METAMVGL, a multi-view graph-based contig binning algorithm to integrate both assembly and PE graphs to label short contigs and correct initial labeling errors. PE graph could link the dead ends to the main graph and increase the graph connectivity. METAMVGL automatically weights the two graphs and performs label propagation to label the short contigs. In experiments, we observed METAMVGL could substantially improve the recalls without loosing any precision comparing with the existing contig binning tools, especially for the metagenomic data from the complex microbial community (Fig. 4c, f). We also evaluated METAMVGL: 1. on the assembly graphs from metaSPAdes and MEGAHIT; and 2. using the initial binning labels from different tools. All these results support METAMVGL outperform GraphBin in different experimental configurations. On average, METAMVGL could finish the contig binning in 3.38 min and requires 2.81 Gb RAM to store the two graphs and perform label propagation. It requires a little bit more computational resources than GraphBin due to the analysis of more complex and complete graph (Additional file 7). Sometimes, we found the combined graph was still incomplete after incorporating both assembly and PE graphs and there still required to consider other information to reveal contig long-range connectivity from various long-fragment sequencing (PacBio and Oxford Nanopore sequencing) or linked-read sequencing (Tell-seq and stLFR sequencing) technologies.

## Conclusion

Metagenomic sequencing has been proved as an efficient technology to explore and recognize the novel microbes in the environmental and human fecal samples. Due to the scarcity of the reference genomes, the genomes of novel species could be obtained by de novo assembly. Because only fragmented contigs can be assembled from the mainstream short-read sequencing technologies, the interests rise quickly in developing efficient contig binning algorithms. But most of the available algorithms can only handle long contigs based on their sequence contexts and read depths. In this study, we developed METAMVGL, a multi-view graph-based contig binning algorithm to integrate both assembly and PE graphs to label short contigs and correct initial labeling errors. METAMVGL could weight the two graphs automatically and connect the dead ends to the main graph efficiently. Our experiments proved it can significantly improve the recalls without loosing any precision comparing with the existing contig binning tools on the metagenomic sequencing data from simulation, mock communities and infant fecal samples. We believe METAMVGL would attract more interests of the fast-growing metagenomic research field and pave the way to future understanding the microbial genome dark matter.

## Supplementary information

**Additional file 1.** The statistics of assemblies from MEGAHIT andmetaSPAdes for the simulated, *BMock12*, *SYNTH64* and *Sharon* datasets.

**Additional file 2.** The statistics of the largest component (main graph) inthe assembly graph, PE graph, and combined graph for the simulated,*BMock12*, *SYNTH64* and *Sharon* datasets.

**Additional file 3.** The performance of MetaBAT2, GraphBin andMETAMVGL on the BMock12, SYNTH64 and Sharon datasets: (a) and(d) for *BMock12* dataset; (b) and (e) for *SYNTH64* dataset; (c) and (f)for *Sharon* dataset. MEGAHIT and metaSPAdes are used to generate theassembly graphs. The initial binning tool is **MetaBAT2**.

**Additional file 4.** The performance of MyCC, GraphBin and METAMVGLon the *BMock12*, *SYNTH64* and *Sharon* datasets: (a) and (d) for*BMock12* dataset; (b) and (e) for *SYNTH64* dataset; (c) and (f) for*Sharon* dataset. MEGAHIT and metaSPAdes are used to generate theassembly graphs. The initial binning tool is **MyCC**.

**Additional file 5.** The performance of CONCOCT, GraphBin andMETAMVGL on the *BMock12*, *SYNTH64* and *Sharon* datasets: (a) and(d) for *BMock12* dataset; (b) and (e) for *SYNTH64* dataset; (c) and (f)for *Sharon* dataset. MEGAHIT and metaSPAdes are used to generate theassembly graphs. The initial binning tool is **CONCOCT**.

**Additional file 6.** The performance of SolidBin, GraphBin and METAMVGLon the *BMock12*, *SYNTH64* and *Sharon* datasets: (a) and (d) for*BMock12* dataset; (b) and (e) for *SYNTH64* dataset; (c) and (f) for*Sharon* dataset. MEGAHIT and metaSPAdes are used to generate theassembly graphs. The initial binning tool is **SolidBin**.

**Additional file 7.** Running time and memory usage of GraphBin andMETAMVGL.

## Data Availability

The source code of METAMVGL is publicly available at https://github.com/ZhangZhenmiao/METAMVGL. The Illumina short-reads of *BMock12*, *SYNTH64* and *Sharon* datasets are available in NCBI Sequence Read Archive (SRA); the accession numbers are SRX4901583, SRX200676 and SRX144807, respectively. This article has been published as part of BMC Bioinformatics Volume 22 Supplement 10 2021: Selected articles from the 19th Asia Pacific Bioinformatics Conference (APBC 2021): bioinformatics. The full contents of the supplement are available at https://bmcbioinformatics.biomedcentral.com/articles/supplements/volume-22-supplement-10.

## Abbreviations

METAMVGL: Metagenomic contig binning using multi-view graph learning
PE graph: Paired-end graph
PE link: Paired-end link
TNF: Tetranucleotide frequencies
ARI: Adjusted rand index
RAM: Random-access memory

## References

[1] Li J, Jia H, Cai X, Zhong H, Feng Q, Sunagawa S, Arumugam M, Kultima JR, Prifti E, Nielsen T (2014). An integrated catalog of reference genes in the human gut microbiome. Nat Biotechnol.

[2] Truong DT, Franzosa EA, Tickle TL, Scholz M, Weingart G, Pasolli E, Tett A, Huttenhower C, Segata N (2015). Metaphlan2 for enhanced metagenomic taxonomic profiling. Nat Methods.

[3] Almeida A, Mitchell AL, Boland M, Forster SC, Gloor GB, Tarkowska A, Lawley TD, Finn RD (2019). A new genomic blueprint of the human gut microbiota. Nature.

[4] Almeida A, Nayfach S, Boland M, Strozzi F, Beracochea M, Shi ZJ, Pollard KS, Sakharova E, Parks DH, Hugenholtz P, et al. A unified catalog of 204,938 reference genomes from the human gut microbiome. Nat Biotechnol. 2020; 1–10. 10.1038/s41587-020-0603-3 PMC7801254 32690973

[5] Poyet M, Groussin M, Gibbons S, Avila-Pacheco J, Jiang X, Kearney S, Perrotta A, Berdy B, Zhao S, Lieberman T (2019). A library of human gut bacterial isolates paired with longitudinal multiomics data enables mechanistic microbiome research. Nat Med.

[6] Nayfach S, Shi ZJ, Seshadri R, Pollard KS, Kyrpides NC (2019). New insights from uncultivated genomes of the global human gut microbiome. Nature.

[7] Zou Y, Xue W, Luo G, Deng Z, Qin P, Guo R, Sun H, Xia Y, Liang S, Dai Y (2019). 1520 reference genomes from cultivated human gut bacteria enable functional microbiome analyses. Nat Biotechnol.

[8] Forster SC, Kumar N, Anonye BO, Almeida A, Viciani E, Stares MD, Dunn M, Mkandawire TT, Zhu A, Shao Y (2019). A human gut bacterial genome and culture collection for improved metagenomic analyses. Nat Biotechnol.

[9] Consortium HMJRS (2010). A catalog of reference genomes from the human microbiome. Science.

[10] Pasolli E, Asnicar F, Manara S, Zolfo M, Karcher N, Armanini F, Beghini F, Manghi P, Tett A, Ghensi P (2019). Extensive unexplored human microbiome diversity revealed by over 150,000 genomes from metagenomes spanning age, geography, and lifestyle. Cell.

[11] Wu Y-W, Simmons BA, Singer SW (2016). Maxbin 2.0: an automated binning algorithm to recover genomes from multiple metagenomic datasets. Bioinformatics.

[12] Kang DD, Li F, Kirton E, Thomas A, Egan R, An H, Wang Z (2019). Metabat 2: an adaptive binning algorithm for robust and efficient genome reconstruction from metagenome assemblies. PeerJ.

[13] Alneberg J, Bjarnason BS, De Bruijn I, Schirmer M, Quick J, Ijaz UZ, Lahti L, Loman NJ, Andersson AF, Quince C (2014). Binning metagenomic contigs by coverage and composition. Nat Methods.

[14] Lin H-H, Liao Y-C (2016). Accurate binning of metagenomic contigs via automated clustering sequences using information of genomic signatures and marker genes. Sci Rep.

[15] Wang Z, Wang Z, Lu YY, Sun F, Zhu S (2019). Solidbin: improving metagenome binning with semi-supervised normalized cut. Bioinformatics.

[16] Yu G, Jiang Y, Wang J, Zhang H, Luo H (2018). Bmc3c: binning metagenomic contigs using codon usage, sequence composition and read coverage. Bioinformatics.

[17] Mallawaarachchi V, Wickramarachchi A, Lin Y (2020). Graphbin: refined binning of metagenomic contigs using assembly graphs. Bioinformatics.

[18] Nie F, Li J, Li X, et al. Parameter-free auto-weighted multiple graph learning: a framework for multiview clustering and semi-supervised classification. In: IJCAI, 2016; p. 1881–7.

[19] Nurk S, Meleshko D, Korobeynikov A, Pevzner PA (2017). metaspades: a new versatile metagenomic assembler. Genome Res.

[20] Li D, Liu C-M, Luo R, Sadakane K, Lam T-W (2015). Megahit: an ultra-fast single-node solution for large and complex metagenomics assembly via succinct de Bruijn graph. Bioinformatics.

[21] Li H. Aligning sequence reads, clone sequences and assembly contigs with bwa-mem. arXiv preprint arXiv:1303.3997 (2013).

[22] Bishara A, Moss EL, Kolmogorov M, Parada AE, Weng Z, Sidow A, Dekas AE, Batzoglou S, Bhatt AS (2018). High-quality genome sequences of uncultured microbes by assembly of read clouds. Nat Biotechnol.

[23] Fritz A, Hofmann P, Majda S, Dahms E, Dröge J, Fiedler J, Lesker TR, Belmann P, DeMaere MZ, Darling AE (2019). Camisim: simulating metagenomes and microbial communities. Microbiome.

[24] Sevim V, Lee J, Egan R, Clum A, Hundley H, Lee J, Everroad RC, Detweiler AM, Bebout BM, Pett-Ridge J (2019). Shotgun metagenome data of a defined mock community using oxford nanopore, pacbio and illumina technologies. Sci data.

[25] Shakya M, Quince C, Campbell JH, Yang ZK, Schadt CW, Podar M (2013). Comparative metagenomic and rrna microbial diversity characterization using archaeal and bacterial synthetic communities. Environ Microbiol.

[26] Sharon I, Morowitz MJ, Thomas BC, Costello EK, Relman DA, Banfield JF (2013). Time series community genomics analysis reveals rapid shifts in bacterial species, strains, and phage during infant gut colonization. Genome Res.

[27] Wood DE, Lu J, Langmead B (2019). Improved metagenomic analysis with kraken 2. Genome Biol.

