# Supplementary material for: METAMVGL: a multi-view graph-based metagenomic contig binning algorithm by integrating assembly and paired-end graphs
Source: BMC Bioinformatics. 2021 Jul 22;22(Suppl 10):378. doi: 10.1186/s12859-021-04284-4 (PMC8296540; doi:10.1186/s12859-021-04284-4)
Supplement: Supplementary file 4 — Additional file 4. The performance of MyCC, GraphBin and METAMVGLon the BMock12, SYNTH64 and Sharon datasets: (a) and (d) forBMock12 dataset; (b) and (e) for SYNTH64 dataset; (c) and (f) forSharon dataset. MEGAHIT and metaSPAdes are used to generate theassembly graphs. The initial binning tool is MyCC. [file 12859_2021_4284_MOESM4_ESM.pdf]

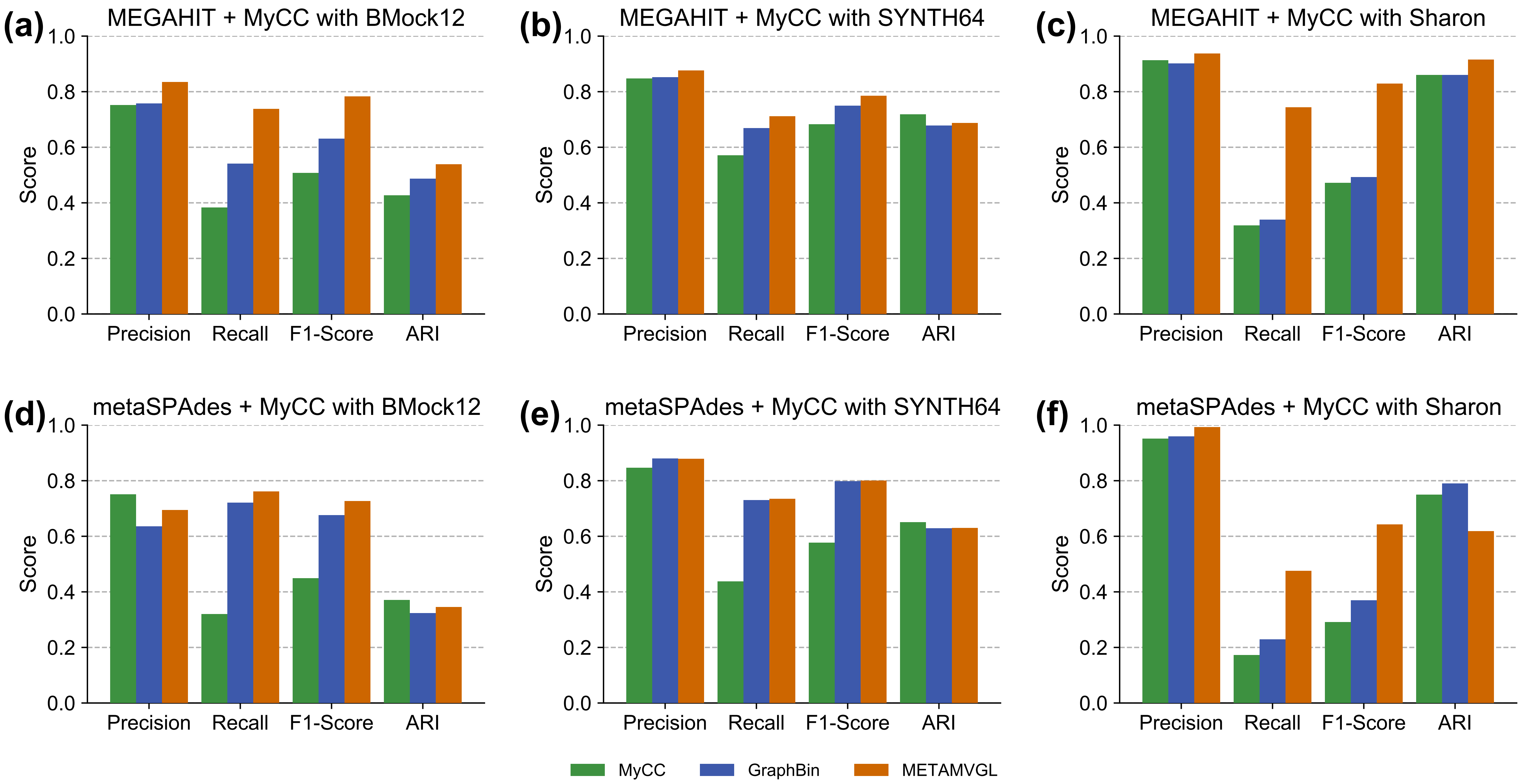
(a)
MEGAHIT + MyCC with BMock12
(b)
MEGAHIT + MyCC with SYNTH64
(c)
MEGAHIT + MyCC with Sharon
(d)
metaSPAdes + MyCC with BMock12
(e)
metaSPAdes + MyCC with SYNTH64
(f)
metaSPAdes + MyCC with Sharon
Score
0.0
0.2
0.4
0.6
0.8
1.0
Precision
Recall
F1-Score
ARI
MyCC
GraphBin
METAMVGL
