# Supplementary material for: METAMVGL: a multi-view graph-based metagenomic contig binning algorithm by integrating assembly and paired-end graphs
Source: BMC Bioinformatics. 2021 Jul 22;22(Suppl 10):378. doi: 10.1186/s12859-021-04284-4 (PMC8296540; doi:10.1186/s12859-021-04284-4)
Supplement: Supplementary file 5 — Additional file 5. The performance of CONCOCT, GraphBin andMETAMVGL on the BMock12, SYNTH64 and Sharon datasets: (a) and(d) for BMock12 dataset; (b) and (e) for SYNTH64 dataset; (c) and (f)for Sharon dataset. MEGAHIT and metaSPAdes are used to generate theassembly graphs. The initial binning tool is CONCOCT. [file 12859_2021_4284_MOESM5_ESM.pdf]

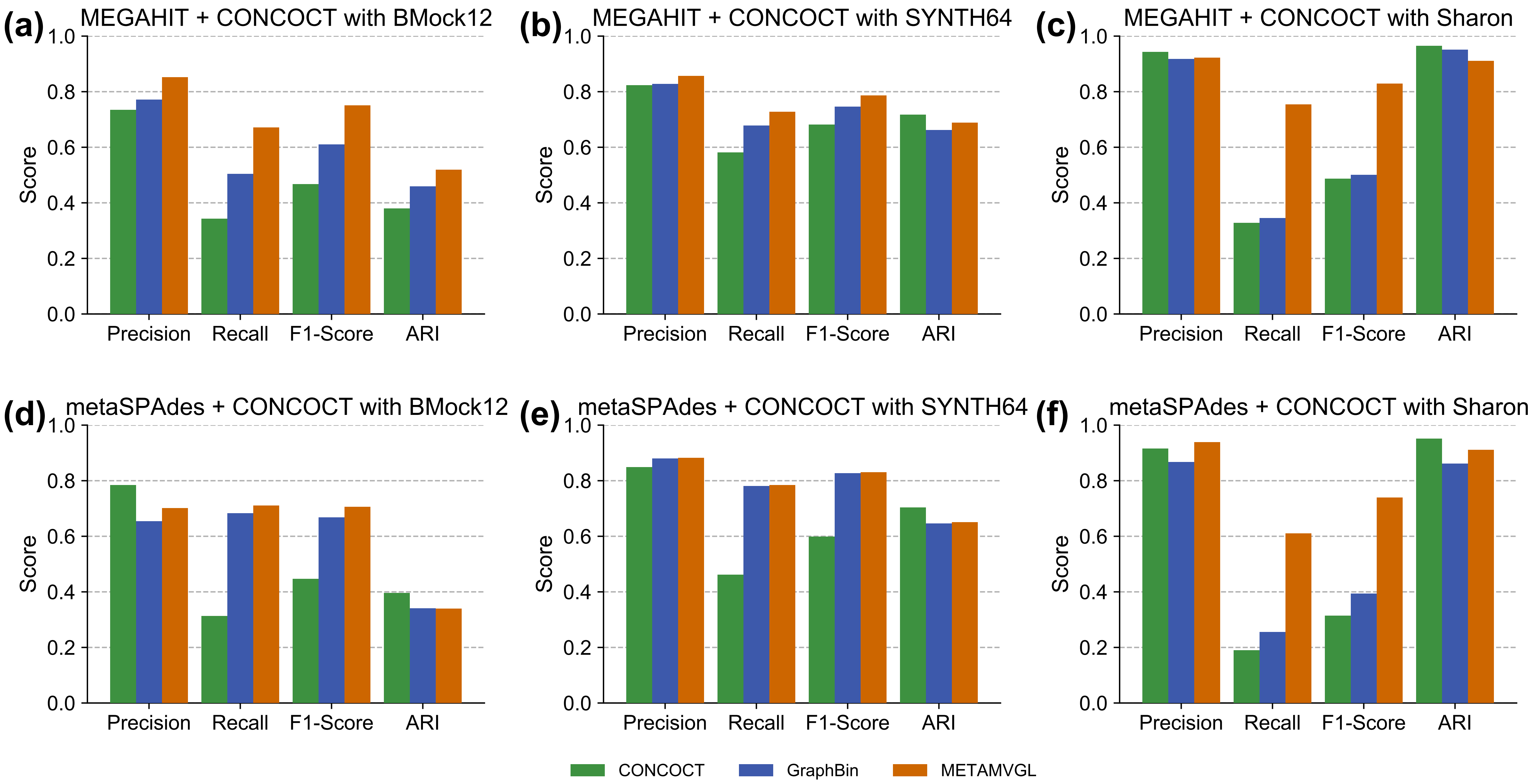

(a) MEGAHIT + CONCOCT with BMock12
(b) MEGAHIT + CONCOCT with SYNTH64
(c) MEGAHIT + CONCOCT with Sharon
(d) metaSPAdes + CONCOCT with BMock12
(e) metaSPAdes + CONCOCT with SYNTH64
(f) metaSPAdes + CONCOCT with Sharon
Score
0.0
0.2
0.4
0.6
0.8
1.0
Precision
Recall
F1-Score
ARI
CONCOCT
GraphBin
METAMVGL
